# Quantitative trait loci for sensitivity to ethanol intoxication in a C57BL/6J × 129S1/SvImJ inbred mouse cross

**DOI:** 10.1007/s00335-012-9394-2

**Published:** 2012-02-28

**Authors:** Elissa J. Chesler, Aaron Plitt, Daniel Fisher, Benita Hurd, Lauren Lederle, Jason A. Bubier, Carly Kiselycznyk, Andrew Holmes

**Affiliations:** 1 The Jackson Laboratory, 600 Main St, Bar Harbor, ME 04609 USA; 2 Laboratory of Behavioral and Genomic Neuroscience, National Institute on Alcoholism and Alcohol Abuse (NIAAA), NIH, Bethesda, MD USA

## Abstract

Individual variation in sensitivity to acute ethanol (EtOH) challenge is associated with alcohol drinking and is a predictor of alcohol abuse. Previous studies have shown that the C57BL/6J (B6) and 129S1/SvImJ (S1) inbred mouse strains differ in responses on certain measures of acute EtOH intoxication. To gain insight into genetic factors contributing to these differences, we performed quantitative trait locus (QTL) analysis of measures of EtOH-induced ataxia (accelerating rotarod), hypothermia, and loss of righting reflex (LORR) duration in a B6 × S1 F2 population. We confirmed that S1 showed greater EtOH-induced hypothermia (specifically at a high dose) and longer LORR compared to B6. QTL analysis revealed several additive and interacting loci for various phenotypes, as well as examples of genotype interactions with sex. QTLs for different EtOH phenotypes were largely non-overlapping, suggesting separable genetic influences on these behaviors. The most compelling main-effect QTLs were for hypothermia on chromosome 16 and for LORR on chromosomes 4 and 6. Several QTLs overlapped with loci repeatedly linked to EtOH drinking in previous mouse studies. The architecture of the traits we examined was complex but clearly amenable to dissection in future studies. Using integrative genomics strategies, plausible functional and positional candidates may be found. Uncovering candidate genes associated with variation in these phenotypes in this population could ultimately shed light on genetic factors underlying sensitivity to EtOH intoxication and risk for alcoholism in humans.

**Electronic supplementary material:**

The online version of this article (doi:10.1007/s00335-012-9394-2) contains supplementary material, which is available to authorized users.

## Introduction

Multiple factors influence the propensity to consume alcohol and the risk for developing an alcohol use disorder. Of these, decreased sensitivity to acute alcohol challenge has been found to be a predictor of risk for alcohol abuse (Newlin and Thomson 1990; Schuckit 1994). Increased sensitivity to the unpleasant subjective effects of intoxication, such as ataxia and sedation, has been posited to serve as a protective influence by discouraging drinking (Krystal et al. 2003). However, the relationship between sensitivity and drinking holds in some, but not all, cases of altered ethanol (EtOH)-related behaviors in various rodent stocks (reviewed in Crabbe et al. 2006). Nonetheless, understanding the neurobiological basis of sensitivity could provide insight into the etiology and pathophysiology of alcohol abuse.

Since the observation that inbred mouse strains exhibit marked differences in voluntary EtOH consumption (e.g., Belknap et al. 1993; McClearn and Rodgers 1959), inbred mice have been utilized as a tool to study the genetics of multiple alcohol-related phenotypes, including sensitivity to intoxication (e.g., Bachmanov et al. 2002; Crabbe 1983; Crabbe et al. 2005; Kakihana et al. 1966; Milner and Buck 2010; Tabakoff et al. 2008). However, the underlying genetics of these traits is still not well understood despite the increasing availability of techniques for studying gene–phenotype relationships.

In this context, we previously reported that two inbred mouse strains, C57BL/6J (hereafter abbreviated B6) and 129S1/SvImJ (hereafter abbreviated S1), differ markedly in sensitivity to acute EtOH intoxication (Chen and Holmes 2009; Palachick et al. 2008). In these studies, this difference in sensitivity was evidenced by increased loss of righting reflex (LORR) responses in S1, relative to B6, in response to a moderate–high dose (3 g/kg) of EtOH. By contrast, B6 and S1 did not vary in hypothermic responses to the same (3 g/kg) dose or in ataxia responses to a 1.75 g/kg dose, consistent with a specific pharmacodynamic, rather than general pharmacokinetic, difference between the two strains. These data are generally consistent with the findings of Crabbe and colleagues obtained from a larger inbred strain comparison in which the authors also observed relatively greater responses in S1 than in B6 on various EtOH behaviors (Crabbe et al. 2003a, b, 2005; Metten et al. 2004; Rustay et al. 2003).

Quantitative trait locus (QTL) analysis has been employed as a useful approach to leveraging strain differences to uncover genetic influences underlying variation in alcohol-related phenotypes (Plomin and McClearn 1993). The discovery of QTLs associated with such traits provides a foundation for the identification of specific candidate genes (Shirley et al. 2004). These candidates are likely of relevance to genes underlying variation in alcohol-related behaviors and risk for alcoholism in human populations (Ehlers et al. 2010). Because verification and refinement of such QTLs is facilitated by comparison across different intercrossed populations, the aim of the current study was to employ this approach in order to identify QTLs associated with variation in sensitivity to acute EtOH challenge in a novel F2 population of B6 × S1 mice. We examined the population on multiple phenotypes (ataxia, hypothermia, and LORR) given evidence that different behavioral end-point measures of sensitivity are genetically dissociable (Crabbe et al. 1996, 2005).

## Materials and methods

### General procedures

A battery of three assays for intoxication was employed: EtOH-induced ataxia, hypothermia, and LORR. All mice were tested on each assay with the assay involving the lowest dose (i.e., ataxia) first, followed by hypothermia and then LORR, with an interval of at least 1 week between tests. Long-term tolerance to EtOH’s effects was not expected to occur with this test and treatment regimen (Crabbe 2007). For all assays, EtOH (200 proof, prepared in 0.9% saline to produce 20% v/v solutions) was administered via intraperitoneal (i.p.) injection with dose determined by manipulating the volume of injection.

These and all experimental procedures were approved by the National Institute on Alcohol Abuse and Alcoholism Animal Care and Use Committee and strictly followed the NIH guidelines “Using Animals in Intramural Research.”

### EtOH-induced ataxia

The accelerating rotarod was used to test for EtOH-induced ataxia, using procedures described previously (Hefner and Holmes 2007). The apparatus used was a Med Associates rotarod typically used for testing rats (model ENV-577, Med Associates, St. Albans, VT), with a 7-cm-diameter dowel covered with 320-grit sandpaper providing a uniform surface that prevented gripping the dowel, as recommended to improve the validity of the assay (Rustay et al. 2003). Mice were placed onto the rotarod dowel which was then accelerated at a constant rate of 8 rpm/min up to 40 rpm. Latency to fall to the floor 10.5 cm below was automatically recorded by photocell beams, with a maximum cutoff latency of 5 min. Mice first received ten consecutive training trials separated by a 30 s intertrial interval. The average latency to fall over the ten training trials was calculated. In addition, the change in latency to fall from the first to the last trial (called the training index) was taken as a measure of motor learning.

Twenty-four hours after training, mice were given an acclimation trial followed by two more pre-EtOH trials that were averaged to establish pre-EtOH performance, and then they were injected with 2.0 g/kg EtOH. Thirty minutes later there was an acclimation trial followed by two test trials that were averaged to get the post-EtOH performance. The dependent measure was the difference between the pre- and post-EtOH performance averages, called the ataxia index.

### EtOH-induced hypothermia

EtOH-induced hypothermia was tested as previously described (Boyce-Rustay et al. 2008b) in a room with an ambient temperature of 23°C. Basal core body temperature was taken by inserting a Thermalert TH-5 thermometer (Physitemp, Clifton, NJ) 2 cm into the rectum until a stable reading was obtained. Mice were then injected with 3.5 g/kg EtOH and temperature was measured 30, 60, 90, and 120 min later. The difference (delta temperature) between pre-EtOH temperature and the average temperature over the 4 post-EtOH time points was taken as the dependent measure.

### EtOH-induced LORR

EtOH-induced LORR was assessed using methods described previously (Daws et al. 2006). Mice were injected with 3.5 g/kg EtOH and immediately placed into the supine position in a V-shaped chamber. LORR duration was measured as the time from injection to recovery of the righting reflex (i.e., turning onto all four paws twice in 30 s after initial self-righting), with a maximum latency of 180 min before the experiment was terminated.

At LORR recovery mice were killed via cervical dislocation and rapid decapitation. Trunk blood was taken for analysis of blood EtOH concentrations (BECs) using the Analox AM1 Alcohol Analyzer (Analox Instruments USA Inc., Lunenburg, MA).

### Parental strain dose-response comparison

As noted in the Introduction, we previously reported that B6 and S1 differ in the LORR response to 3 mg/kg EtOH, but not in either the hypothermia response to 3 mg/kg EtOH or the ataxic response to 1.75 g/kg EtOH (Chen and Holmes 2009; Palachick et al. 2008). To confirm these differences and extend the strain comparison to higher doses, we compared male B6 and S1, obtained from The Jackson Laboratory (Bar Harbor, ME), for responses in each assay to two different EtOH doses: ataxia (1.75 or 2.0 mg/kg), hypothermia (3.0 or 3.5 mg/kg), and LORR (3.0 or 3.5 mg/kg) (*n* = 8 per strain, per dose). Other than dose, procedures were as described above.

In addition, to exclude potential EtOH pharmacokinetic differences between S1 and B6, EtOH metabolism was assessed in a separate cohort of EtOH-naïve mice by measuring BECs at various time points following injection with 3.5 g/kg EtOH. Specifically, BECs were measured 5, 30, 60, and 240 min following injection of 3.5 g/kg EtOH, as described previously (Boyce-Rustay and Holmes 2006). To avoid trauma to any single region and conform to local ACUC regulations, blood samples were taken from the submandibular vein at 5 and 30 min, from the tail at 60 min, and from the trunk (after rapid cervical dislocation and rapid decapitation) at 240 min. BECs were measured using the Analox AM1 Alcohol Analyzer.

### F2 phenotype

F1 mice were bred in-house from B6 and S1 mice obtained from The Jackson Laboratory. F2 mice were bred in-house from 16 F1 × F1 breeding pairs. We generated and analyzed 346 F2 mice (183 males, 160 females) derived from 39 separate litters. For comparison with the F2, 11 B6, 8 S1 (obtained from The Jackson Laboratory), and 8 F1 (bred in-house) male mice were tested concomitantly with the F2 mice.

Mice were group-housed by sex and litter in a temperature- and humidity-controlled vivarium under a 12-h light/dark cycle (lights on 0600 h) with ad libitum access to food and water. Testing began when mice were at least 2 months of age, with a test range of 2–8 months necessitated by the practicalities of testing a large number of mice. Potential age (or litter) effects were not systemically tested for. Note that all F1 and F2 mice had been previously tested (data unpublished) for Pavlovian fear extinction (procedure as in Camp et al. 2009). Our goal was to fully utilize the generation of this large F2 population by examining two phenotypic domains (fear and EtOH sensitivity) in which B6 and S1 differ markedly (Palachick et al. 2008; Whittle et al. 2010).

### Statistical analysis of phenotype data

The effect of the training trial on rotarod latency to fall was analyzed using repeated-measures analysis of variance (ANOVA). The effect of strain on EtOH-induced ataxia, hypothermia, LORR, and BECs at recovery was analyzed using ANOVA followed by Fisher’s Least-Significant Difference post-hoc tests. The effect of strain and time-point effects on BECs was analyzed using ANOVA, with repeated measures for time point.

### Genotyping analysis

Tail samples were obtained from F2 mice and shipped to the Cancer Animal Models Core Facility at Emory University School of Medicine (Atlanta, GA) for processing and analysis. Tail samples were lysed overnight in standard proteinase K buffer and then purified by bead extraction using the Biorobot M48 system (Qiagen, Valencia, CA). DNA samples were resuspended and DNA concentration determined by picogreen analysis (SpectraMax XPS, Molecular Devices, Sunnyvale, CA). Purified DNA samples were analyzed utilizing the Murine Medium Density Linkage Panel (Illumina Inc., San Diego, CA) using the manufacturer’s standard protocol. Briefly, 250 ng of purified DNA was subjugated to analysis and then loaded onto 32 sample beadchips. Beadchips were assayed on the Illumina Beadarray reader and then analyzed using the manufacturer’s software. Resultant data were imported into Excel (Microsoft Corp., Redmond, WA) for manual correction of implausible recombinations, poor clustering of alleles, and other evidence of bad marker performance. The file was then formatted for input to R/QTL. Of the 1,449 SNPs on the LD panel, 880 (61%) differed between the parental strains and were thus informative. Of these, 878 (99.7%) gave the expected call with parental control DNA. In addition, two samples (Nos. 23 and 141) were repeated in independent experiments and were shown to demonstrate >99% identity of allele calls between the two separate analytical runs.

### QTL mapping

QTL mapping was performed using the R/QTL package (Broman et al. 2003; Manichaikul et al. 2009). Six phenotypic measures were subject to QTL analysis: rotarod baseline, rotarod learning, rotarod ataxia, hypothermia, LORR, and BECs at LORR recovery. In each case, a main-effects (single-locus) scan was first applied to find suggestive and significant main effects. Additive and interacting sex co-factors were then analyzed to search for sex-specific loci. Each single-locus scan of autosomes was subject to 1,000 permutations, with a separate permutation for the X chromosome to determine significance thresholds. Finally, a pairwise QTL scan, which included additive and interacting cofactors, was performed with 250 permutations. Suggestive main-effects loci (*P* < 0.63) and suggestive pairwise loci (full model *P* < 0.63) were included in a multiple-QTL model, which included an additive and fully interacting effect of sex. The models were reduced by backward elimination using the stay criterion of *P* < 0.05 for each term.

The 1.5-LOD confidence interval was identified using R/QTLs lodint function. QTLs for other alcohol-related measures were found by querying the Mouse Genome Database (MGD) for any QTLs within this confidence interval. All positional candidates for loci mapped in the present study and those found in the MGD were imported into the GeneWeaver software system (Baker et al. 2011), which enables discovery of hierarchical intersections among gene set-centered data. We compared positional candidates with other functional genomics data sets, including 39 sets of genes from differential expression and coexpression studies, using GeneWeaver’s “PhenomeMap” function. This enabled us to identify high-order intersections among gene sets, including the set of QTL positional candidates at a given locus.

## Results

### Parental strain dose-response comparison

**Fig. 1:**
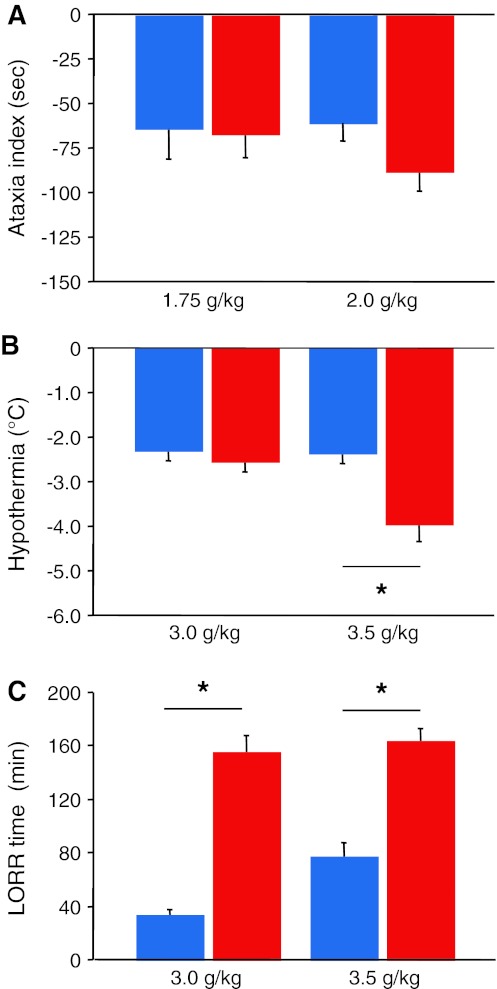
Trait differences between parental strains. **a** S1 (*red bars*) and B6 (*blue bars*) mice did not differ in ataxia responses to either 1.75 or 2.0 g/kg EtOH. **b** S1 had a significantly greater hypothermia response than B6 to a 3.5- but not a 3.0-g/kg EtOH dose. **c** S1 had significantly longer LORR responses than B6 to either a 3.0- or a 3.5-g/kg EtOH dose. *P < 0.05. Data are mean ± SEM (colour figure online)

B6 and S1 did not significantly differ in the average rotarod training latency or changes in latency across training trials (data not shown). Strain comparison also found no significant effect of strain or EtOH dose (or interaction between the two) for ataxia responses to 1.75 or 2.0 g/kg EtOH (Fig. 1a). For hypothermia responses, there was a significant interaction between strain and EtOH dose (*F*_1,28_ = 5.46, *P* < 0.05, *n* = 8/strain/dose). Fisher’s post-hoc tests showed that S1 had a significantly greater hypothermia response than B6 to the 3.5-g/kg but not to the 3.0-g/kg dose (Fig 1b). For LORR, there was also a significant main effect of strain (*F*_1,28_ = 123.49, *P* < 0.01, *n* = 8/strain/dose) and EtOH dose (*F*_1,28_ = 7.57, *P* < 0.01) and near significant interaction between the two (*F*_1,28_ = 3.77, *P* = 0.062). Fisher’s post-hoc tests showed that S1 had significantly longer LORR than B6 to the 3.0- and 3.5-g/kg doses (Fig. 1c).

In a separate cohort tested for EtOH clearance, there was a significant main effect of time point on BECs (*F*_3,33_ = 21.51, *P* < 0.01), but not strain or strain × time interaction, indicating an absence of strain differences and a reduction in BEC values across time points (5 min: S1 = 389 ± 16, B6 = 339 ± 38; 30 min: S1 = 372 ± 18, B6 = 336 ± 18; 60 min: S1 = 306 ± 19, B6 = 299 ± 19; 240 min: S1 = 243 ± 16, B6 = 239 ± 21; *n* = 6–7 per strain).

### F2 phenotype and QTL analysis

Significant or suggestive QTLs were detected and multi-locus models identified for each trait (Table 1). The 1.5 LOD confidence interval was determined for each locus (Table 2).

**Table 1:** Multiple QTL models

Trait	Effect	Peak marker	LOD	%Variance	*F*	*P*
Hypothermia						
	3@80.2	rs3710548	3.607	4.272	4.131	0.002788
	7@18.4	rs13479153	2.561	3.011	5.824	0.003261
	16@30.9	rs4182243	2.708	3.188	6.166	0.002345
	Sex		4.167	4.953	6.387	0.000321
	3@80.2:sex		3.039	3.586	6.935	0.001119
LORR						
	Sex		24.293	25.7219	8.0809	2.55E − 15
	2@21.4	rs13476399	6.785	6.3172	2.4808	0.00417
	4@93.4	rs3695715	3.255	2.9549	3.4812	0.00849
	8@22.5	rs3666140	3.595	3.2715	3.8542	0.00454
	12@31.0	rs6344105	8.0043	7.5183	2.9525	0.00067
	19@33.8	rs6194426	2.6302	2.3771	2.8005	0.02621
	sex:2@21.4		5.2937	4.8763	3.8299	0.00108
	sex:4@93.4		0.4142	0.3685	0.8682	0.42076
	sex:8@22.5		3.3885	3.079	7.2548	0.00084
	sex:12@31.0		4.6929	4.3043	3.3806	0.00307
	sex:19@33.8		2.432	2.1948	5.1715	0.0062
	2@21.4:12@31.0		5.2204	4.8062	2.8311	0.00484
	sex:2@21.4:12@31.0		4.3595	3.9889	4.6994	0.00109
BECs						
	Sex		2.39	2.889	3.571	0.014446
	7@6.3	rs13479145	4.899	6.029	3.726	0.001361
	9@11.6	rs13480854	4.002	4.893	4.535	0.001422
	11@2.6	rs3697686	5.317	6.563	4.056	0.000626
	sex:9@11.6		1.515	1.819	3.373	0.035568
	7@6.3:11@2.6		2.58	3.122	2.894	0.022406
Rotarod average^a^						
	Sex		3.336	3.379	4.965	0.002203
	1@92.7	rs3700831	2.241	2.253	4.966	0.007503
	4@9.0	rs13477617	3.774	3.834	8.45	0.000264
	5@2.8	rs13478110	3.398	3.443	3.794	0.004952
	6@48.2	rs6239023	3.74	3.798	8.37	0.000285
	7@48.4	mCV23423763	2.633	2.655	5.85	0.003187
	12@64.5	rs13481614	2.238	2.25	4.959	0.007556
	18@56.2	rs4137441	1.879	1.884	4.153	0.016549
	sex:5@2.8		3.335	3.378	7.445	0.000688
Training index						
	11@42.4	rs13481076	3.417	4.434		0.000409
Ataxia						
	sex		0.9151	0.9477	3.898	0.049212
	8@39.7	rs3699406	9.6403	10.586	2.419	0.001172
	9@44.0	rs3655717	10.2343	11.2837	2.578	0.000506
	17@20.2	rs3672987	11.406	12.676	2.896	9.05E-05
	8@39.7:9@44.0		4.4048	4.6692	1.6	0.090041
	8@39.7:17@20.2		6.0238	6.4552	2.212	0.011097
	9@44.0:17@20.2		6.7097	7.2235	2.476	0.004152
	8@39.7:9@44.0:17@20.2		3.9876	4.2152	2.167	0.029703

Effect sizes for the peak marker(s), LOD scores, *F* statistic, and degrees of freedom are given for dropping each term from the model

^a^Due to the large number of loci in the model, multilocus interactions were not tested extensively

**Table 2:** Proportional hazards mapping for censored trait data

Trait	Marker	Chr	Position (Mb)	LOD	LOD *P*	LOD *P*μ
Rotarod average^a^	rs6239023	6	94.005991	4.03334	0.266541	3.766629
	rs4226783	7	100.081465	3.820091	0.691785	3.129
	rs3719581	11	86.772383	4.824871	0.251099	4.573533
	rs3702256	X	131.483758	3.133643	0.336518	2.796845
LORR	rs6268364	4	151.390225	5.74	4.853	0.884
	rs3718735	6	101072507	3.95	1.287	2.665

^a^Due to the censored data distribution, multilocus interactions were not tested extensively

#### EtOH-induced hypothermia

**Fig. 2:**
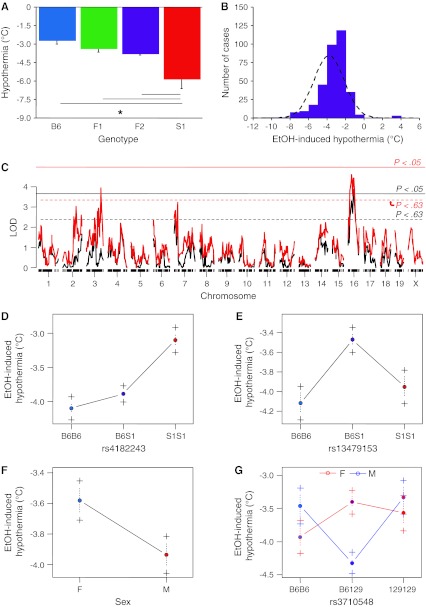
EtOH-induced hypothermia. **a** S1 had a greater EtOH-induced hypothermia response than B6, F1, and F2. **b** *Frequency histogram* showing approximately normal distribution (*dashed line* is normal distribution) in the F2 population. **c** A scan for single-locus main effects found a significant locus on Chr 16 (not shown), which was also detected using additive (*black LOD trace*) and interacting (*red LOD trace*) sex covariates, though the effect was merely suggestive with a covariate in the model. Additional loci were found on Chr 7 with an additive sex covariate and on Chr 3 with an interacting sex covariate. The empirical significance threshold *P* < 0.05 for the scan with an additive sex covariate is indicated by a *solid black line*. Suggestive thresholds are indicated by *dashed lines* for the additive sex covariate (*black*) and interacting sex covariate (*red*). **d** Allelic effects for the loci are consistent with a negative dominance deviation of the Chr 16 locus (rs4182243), whereas the Chr 7 locus (rs13479153) is overdominant, and the effects of Chr 3 (rs3710548) are sex dependent such that male heterozygotes have greater hypothermia than homozygotes of either sex and female heterozygotes have less hypothermia. Overall, in the F2 population males had greater EtOH-induced hypothermia scores than females (note that data obtained in the *parental lines* were from males only, precluding direct comparison with this QTL effect); *P < 0.05. Data are mean ± SEM (colour figure online)

Comparison of the B6, S1, F1, and F2 population means revealed differences in hypothermia (*F*_3,368_ = 62.42, *P* < 0.01, *n* = 8–345). Fisher’s post-hoc tests revealed that hypothermia in S1 was greater than that in the three other genotypes (which did not differ from each other) (Fig. 2a). F2 hypothermia scores were quite narrowly distributed around approximately –3°C (Fig. 2b).

QTL analysis and multilocus modeling (Table 1) revealed two significant main-effect loci (*P* < 0.05), as well as a significant sex effect and a third main-effect locus that interacted with sex (Fig. 2c). The first locus was on Chromosome (Chr) 16 at 30.9 cM (LOD = 3.9, *P* < 0.05), with a peak marker at rs4182243 and a 1.5-LOD drop confidence interval from rs4165065 (8.13 cM) to rs4200124 (40.0 cM) that was independent of sex (Fig. 2d). A second locus was detectable with an additive sex effect in the model on Chr 7 at 18.4 cM, with a peak marker at rs13479153 (LOD = 2.8, *P* < 0.01) (Fig. 2e). The third locus, detectable with additive and interacting effects of sex, was found on Chr 3 at 80.2 cM, with a peak marker at rs3710548 (LOD = 3.9, *P* < 0.6) (Fig. 2f, g). Multilocus modeling revealed that together the loci and their interactions accounted for 13.1% of the variance in EtOH-induced hypothermia (Table 1).

#### EtOH-induced LORR and BECs at recovery

**Fig. 3:**
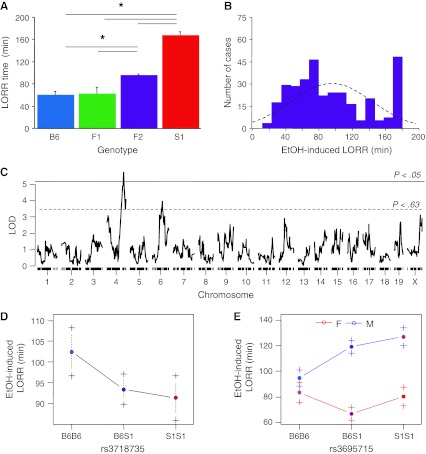
EtOH-induced LORR. **a** EtOH-induced LORR in S1 was greater than that in B6, F1, or F2, and greater in F2 than that in B6. **b** F2 LORR scores were somewhat bimodally distributed (*dashed line* is normal distribution), with many values around 60 min and another cluster around the cutoff of 180 min. **c** A scan for single-locus main effects using the two-part model found a significant main-effect locus on Chr 4 and a suggestive locus on Chr 6. **d** The locus has an S1 dominant effect. **e** Regression revealed a sex difference in the allelic effect of this locus such that males with the S1 alleles had the highest LORR duration; *P < 0.05. Data are mean ± SEM

Comparison of the B6, S1, F1, and F2 population trait means found differences in loss of righting reflex (LORR) time (*F*_3,350_ = 9.44, *P* < 0.01, *n* = 8–327). Fisher’s post-hoc tests revealed that LORR time in S1 was greater than that in the three other genotypes and greater in F2 than in B6 (Fig. 3a). F2 LORR scores were somewhat bimodally distributed, with many values around approximately 60 min and another cluster around the cutoff of 180 min (Fig. 3b). Forty-five F2 mice, representing 12% of the cases, reached the cutoff LORR duration of 180 min.

QTL analysis of LORR required R/QTL’s two-part (proportional hazards) model to account for right-censored phenotypic data (Table 3). This analysis revealed a significant main-effect locus on Chr 4 at 93.4 cM (*P* < 0.05) (Fig. 3c). The Chr 4 locus had a dominant S1 effect (Fig. 3d). The combined LOD for this locus was 5.74 (*P* < 0.05), with a LOD for the proportion censored of 4.853 (*P* < 0.05) and a LOD for the mean difference between genotypes of 0.884 (nonsignificant). A suggestive locus was also detected on Chr 6 at 52.6 cM. The combined LOD for this locus was 3.95 (*P* < 0.63), with a LOD for the mean difference between genotype classes at 2.665 (*P* < 0.63) and a LOD for the difference between proportion censored of 1.287 (nonsignificant). Sex effects and multilocus modeling could be evaluated using only conventional parametric methods in the R/QTL environment, and these results must be interpreted cautiously. The effect of the QTL, which would be downwardly biased in parametric analysis, accounted for 4.8% of the variance in LORR duration. Many suggestive loci and interactions were detected using parametric methods, including a sex interaction with the Chr 4 locus such that males with the S1 alleles had a higher LORR time (Fig. 3e).

**Table 3:** Locations of QTL 1.5 LOD confidence intervals

Peak			Right of peak			Left of peak		
Chr	Marker	Position (Mb)	Flanking	Position (cM)	Position (Mb)	Flanking	Position (cM)	Position (Mb)
Hypothermia								
3	rs3710548	145932289	rs3719390	43.56103	85222358	rs30801216	92.92514	156802752
7	rs13479153	25722935	rs3700068	0	4187548	rs3716088	103.14114	140189839
16	rs4182243	46052770	rs4165065	8.129282	17412172	rs4200124	40.029943	70695141
LORR (normal model)								
2	rs13476399	28144658	rs3713997	0	3151175	rs3679483	104.33488	179861211
4	rs3695715	3649824	rs3663950	71.81746	135285447	rs6279100	103.79191	155557887
8	rs3666140	44049661	rs3661760	8.001332	24557766	rs13479995	64.990121	116236688
12	rs6344105	68860209	rs3706319	26.52421	59053677	rs13481604	61.30464	99317323
19	rs6194426	50203520	rs13483643	27.73783	45386221	rs13483682	38.33064	55236132
LORR (2-part model)								
4	rs6268364	151390225	rs3663950	71.81746	135285447	rs13478068	100.53031	154592281
6	rs3718735	101072507	rs13478783	33.2687	60541373	rs6200835	68.78922	125667502
BECs								
7	rs13479145	19988355	rs6384973	1.031631	5036805	rs3663313	49.842558	63388111
9	rs13480854	7524005	mCV23893269	0	4062079	rs6304156	85.96028	123063108
11	rs3697686	58381052	rs13480836	0	3454200	rs3697686	35.406444	58381052
Rotarod average								
1	rs3700831	177945647	rs6312657	39.70795	69048455	rs13476300	109.1582	192122502
4	rs13477617	26886337	rs3660863	2.380086	7127435	rs3684104	23.498602	38269953
5	rs13478110	9741228	rs13478092	0	3595407	rs3718776	98.87341	150393227
6	rs6239023	94005991	rs3672029	38.74415	75345665	rs30316697	71.27386	130188177
7	mCV23423763	68111945	rs3700068	0	4187548	rs3663988	114.24183	146505067
12	rs13481614	102385663	rs33846822	9.596159	30605487	rs29187760	78.763939	115166913
18	rs4137441	88803388	rs13483426	38.08931	70283358	rs4137441	56.24318	88803388
Training index								
11	rs13481076	66532354	rs3697686	35.40644	58381052	rs3688955	60.98294	90397849
Ataxia								
8	rs3699406	72486070	rs6386110	26.80781	45897379	rs13479995	64.99012	116236688
9	rs3655717	65312971	rs13480112	12.57837	26413932	rs13480421	71.20274	111761261
17	rs3672987	33247165	rs4136382	0	3388912	rs3715723	32.7513	58810428

Physical and genetic locations of the peak marker and flanking markers around the QTL confidence interval, determined by analysis of the one-way scan. For multiple-locus effects, locations are influenced by the other terms in the model and in some cases cannot be readily determined. A large interval should be considered for follow-up studies

**Fig. 4:**
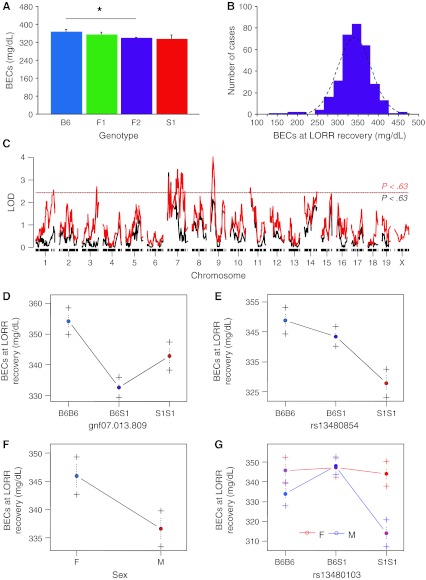
BECs at LORR recovery. **a** BECs at LORR recovery were higher in B6 than in F2. **b** *Frequency histogram* showing largely normal distribution (*dashed line* is normal distribution) in the F2 population. **c** A scan for single-locus main effects found two suggestive loci (*P* < 0.63, *dashed black line*), and a scan for single-locus sex interactions found another suggestive locus on Chr 9. **d–g** Allelic effects of the main-effect loci reveal under dominance on Chr 7 and a slightly positive dominance deviation on Chr 11. The sex difference is such that males had a lower LORR BEC than females, with male S1 homozygotes having the lowest LORR BEC; *P < 0.05. Data are mean ± SEM

There was no significant difference in population means for BECs at LORR recovery (*n* = 6–305). Planned post-hoc tests revealed that BECs were higher in B6 than in F2 (Fig. 4a). BECs in the F2 population were normally distributed around approximately 350 mg/dl (Fig. 4b).

A one-way scan revealed two suggestive main-effect loci for this trait (Fig. 4c). The first locus was on Chr 7 (Fig. 4d) and the second on Chr 11 (Fig. 4e). These loci interacted statistically with each other but not with sex (Fig. 4f). A third locus on Chr 9 was found to interact with sex (Fig. 4c, g). Multilocus modeling estimated that together the loci account for 16.9% of the total phenotypic variance.

#### Motor coordination, learning, and EtOH-induced ataxia

Comparison of the B6, S1, F1, and F2 population means found differences in the average latency to fall across ten trials of rotarod training (*F*_3,351_ = 2.52, *P* < 0.01, *n* = 8–328). Fisher’s post-hoc tests revealed that scores were higher in B6 than in the three other genotypes, while S1 scores were higher than those in F2 (Supplementary Fig. 1A). A frequency histogram of the F2 population indicated a largely normal distribution (Supplementary Fig. 1B). Multiple significant and suggestive main-effect loci were found using a main-effect scan (Table 1, Supplementary Fig. 1C–I), one of which (peak marker rs13478110) interacted with sex (Supplementary Fig. 1J, K). There was also a sex-specific locus on Chr 5 (Supplementary Fig. 1L). These loci were all retained in multiple-locus modeling, together accounting for 25% of phenotypic variance. No higher-order interactions were tested to avoid overfitting the model.

For the rotarod training index there was a trend toward a significant difference in B6, S1, F1, and F2 population means (*F*_3,369_ = 2.36, *P* = 0.072, *n* = 8–346). Planned comparisons post-hoc revealed that S1 showed a greater improvement with training than F1 and F2 (Supplementary Fig. 2A). A frequency histogram of the F2 population indicated a normal distribution, with the majority of scores around 50 s (Supplementary Fig. 2B). A single suggestive main-effect QTL was found (Supplementary Fig. 2C) on Chr 11 at 42 cM (peak marker rs13481076), which accounted for 4.4% of the phenotypic variance.

**Fig. 5:**
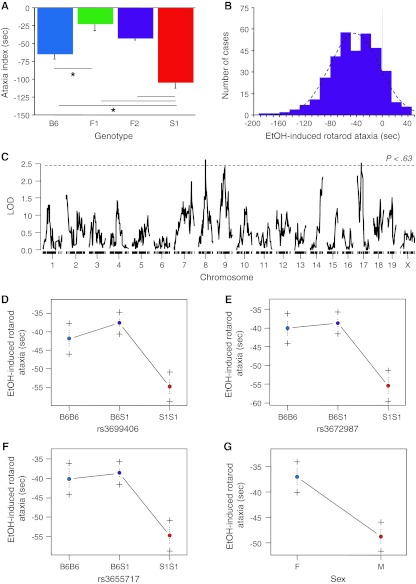
EtOH-induced rotarod ataxia. **a** S1 had a higher-magnitude ataxia index (change in latency to fall from pre-EtOH to post-EtOH trials) than B6, F2, and F1, and B6 had a higher index than F1. **b** *Frequency histogram* illustrates normal distribution (*dashed line* is normal distribution) of training index scores in the F2 population. **c** A scan for single-locus main effects found three suggestive loci at genome-wide *P* < 0.63. **d–g** Main effects of the loci and sex difference, each of which revealed a dominant effect of the B6 allele; *P < 0.05. Data are mean ± SEM

Finally, comparison of the B6, S1, F1, and F2 population means found a significant difference in the EtOH-induced rotarod ataxia index (*F*_3,369_ = 8.96, *P* < 0.01, *n* = 8–346). Fisher’s post-hoc tests revealed that ataxia was greater in S1 than in the other three genotypes and greater in B6 than in F1 (Fig. 5a). F2 ataxia scores were normally distributed around approximately −40 s (Fig. 5B). QTL analysis found three suggestive loci on Chrs 8, 9, and 17 from the main-effect scan (Table 1; Fig. 5c–e). Additional scans and multilocus modeling revealed a main effect of sex (Fig. 5f) and several interactions among the loci, including a three-way interaction (not shown). The main effects of the three loci together accounted for 12.4% of the variance in this phenotype and have allelic effects that mimic the parental differences. Taken together, the sex effects, main effects, and interactions among loci accounted for 22.4% of the phenotypic variance.

### Trait correlations in the F2 population

Table 4 summarizes correlations between phenotypic measures in the F2 population. There were significant, Bonferroni-corrected, negative correlations between average baseline rotarod training latency and EtOH-induced ataxia, between EtOH-induced ataxia and LORR duration, and between LORR duration and BECs at LORR recovery.

**Table 4:** Phenotypic correlations in F2 mice

	Training index	Ataxia	Hypothermia	LORR	BECs
Rotarod average	+0.13	−0.20*	+0.02	−0.16	+0.07
Training index	–	−0.15	+0.05	−0.01	−0.06
Ataxia	–	–	+0.04	−0.33*	−0.06
Hypothermia	–	–	–	−0.12	+0.09
LORR	–	–	–	–	−0.24*

There were significant negative correlations between average baseline rotarod latency and rotarod ataxia, between EtOH-induced rotarod ataxia and LORR duration, and between LORR duration and blood EtOH concentrations (BECs) at LORR recovery

* Bonferroni corrected (*P* < 0.001) significance

### Integrative functional genomics

**Fig. 6:**
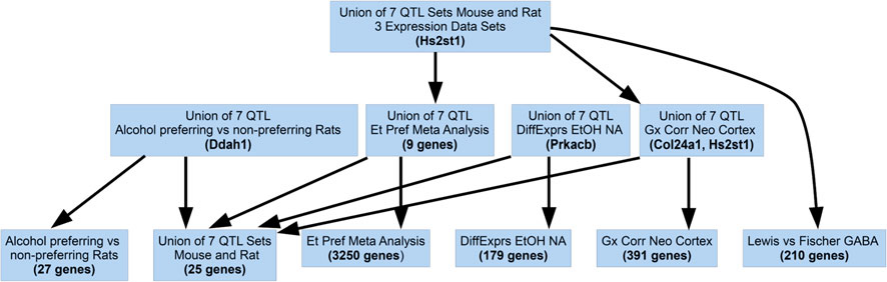
Candidate genes for Chr 3 hypothermia from integrative functional genomics. Hierarchical intersections of functional genomic data with positional candidate genes at six loci that overlap the Chr 3 EtOH hypothermia locus, including four mouse loci and two syntenic loci mapped in rat, were generated using the GeneWeaver Phenome Graph function. *Terminal nodes* represent individual sets of positional candidate genes and published differential expression or coexpression gene sets. *Higher-order nodes* represent two-way, three-way, and higher-order intersections of these sets, respectively. Genes in the highest nodes are connected to the largest number of gene sets and are thus considered more highly supported candidates by empirical evidence. The six QTLs that overlap with the Chr 3 hypothermia QTL are *Alcp3* (Peirce et al. 1998), *Ap6q* (Tarantino et al. 1998), *Letohc1* (Belknap and Atkins 2001), *Lore10* (Bennett et al. 2006) from mouse and *Alcrsp17* (Radcliffe et al. 2006) and *Alcrsp28* (Radcliffe et al. 2009) from rat. The gene expression sets that intersect with positional candidates from the QTL interval are ‘GS128167: Lewis vs. Fischer GABA’ (Sharp et al. 2011) with differential expression in the nucleus accumbens (NA) shell GABA neurons projecting to ventral pallidum in these two strains, ‘GS31783: Gx Corr Neo Cortex’ (Phillips et al. 1994) where the gene expression in BXD Neocortex ILM6v1.1 (Feb08) RankInv microarray data from GeneNetwork.org was correlated with preference for 10% ethanol (g/kg) in a two-bottle choice, ‘GS3647: Et Pref Meta Analysis’ (Mulligan et al. 2006) consisting of genes from the meta-analysis of differential expression in six isogenic and three selected lines with elevated voluntary ethanol consumption, ‘GS87303: Alcohol preferring vs. non-preferring Rats’ (Edenberg et al. 2005) consisting of differential expression in the hippocampus of inbred alcohol-preferring (iP) and -nonpreferring (iNP) rats, and ‘GS128167: DiffExprs EtOH NA’ (Rodd et al. 2008) consisting of differential expression in the NA of inbred alcohol-preferring mice

Several overlapping QTLs were identified among loci in our study and those previously reported in the Mouse Genome Database (Supplementary Table 1). The Chr 3 QTL for EtOH-induced hypothermia overlaps four previously observed QTLs for alcohol preference and consumption. GeneWeaver analysis of these overlapping loci and related data from several functional genomics experiments (Fig. 6) reveals *Hs2st1* as the most highly connected candidate.

## Discussion

The primary aim of the current study was to identify QTLs underlying variation in sensitivity to alcohol intoxication in a F2 B6 × S1 population. We detected a number of loci influencing a set of complex, polygenic phenotypic measures, which in several cases interacted with sex.

The current study was based upon previous observations that the S1 parental strain was significantly more sensitive to the sedative/hypnotic, but not the ataxic or hypothermic, effects of a 3-g/kg dose of EtOH than the B6 parental strain (Chen and Holmes 2009; Palachick et al. 2008; L. DeBrouse et al. (unpublished)). Here, we replicated this difference and further found that a higher EtOH dose (3.5 g/kg) produced not only a greater sedative/hypnotic response but also a greater hypothermic response in S1 compared to B6 mice. We also found that while the strains showed an equivalent ataxic response to a 1.75-g/kg EtOH dose, S1 mice showed modestly greater ataxia to a 2.0-g/kg EtOH dose than B6 that was statistically significant in only one of two experiments. Previous studies using variations on these methods have generally found similar results, including some inconsistency in ataxia measures, in the context of larger inbred strain panels (Crabbe et al. 2003a, b, 2005; Metten et al. 2004; Rustay et al. 2003). Thus, these data confirm our earlier observations that S1 mice are more sensitive to acute EtOH challenge than B6 mice, and extend them by demonstrating that the strains differ across a broader range of measures at increasing EtOH doses.

It is important to note that the current study was conducted in mice that had previously been assessed for Pavlovian fear conditioning and extinction (results to be presented in a future article). To minimize potential carry-over effects, an interval of at least 1 week was interposed between the completion of fear testing and the start of EtOH testing. However, the possibility remains that by virtue of its stressful nature, prior fear testing impacted measures of sensitivity. Discounting, but not fully excluding this possibility, we have previously shown that while sensitivity to EtOH-induced hypothermia and LORR duration was increased in B6 mice by exposure to chronic (14 days) swim stress that ended the day prior to testing, neither acute (1 day) nor subchronic (3 days) stress was sufficient to alter these measures (Boyce-Rustay et al. 2007, 2008a).

The measures of EtOH sensitivity used in the current study cannot dissociate between the initial response to EtOH challenge and acute functional tolerance (AFT) to that response. AFT has a strong genetic component (for review, see Tabakoff et al. 2008). Prior work has shown that S1 mice have a similar AFT as that of B6 to LORR duration (Ponomarev and Crabbe 2004), but have a greater AFT to EtOH-induced ataxia in the rotarod (Rustay and Crabbe 2004) and dowel test (Hu et al. 2008). Greater AFT would be expected to be associated with a decreased, not increased, sensitivity which is opposite to that shown by S1; these data suggest that AFT is unlikely to explain the strain differences. However, more direct examination of AFT in our assays would be necessary to fully exclude a contribution of this process.

**Fig. 7:**
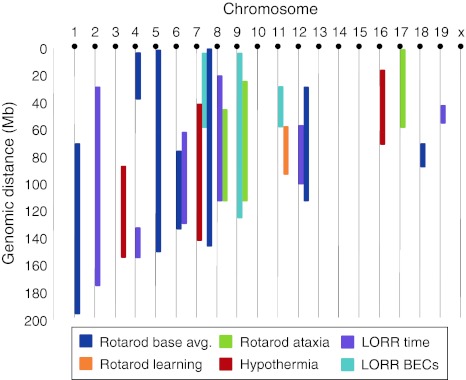
Summary schematic of QTL identified in the current study. QTLs in multilocus models for each phenotype are shown (see *key*). The *bar length* corresponds to the 1.5-LOD confidence interval for each phenotype

QTL analysis was conducted on all three measures of behavioral intoxication as well as pre-EtOH baseline rotarod and motor learning (summarized in Fig. 7). We have the greatest confidence in the QTLs for hypothermia and LORR, which were apparent in a simple main-effect scan. These were found on Chr 16 for hypothermia and on Chrs 4 and 6 for LORR. These QTLs were of generally small effect. While this is typical for QTLs for behavioral traits, it does suggest a significant nongenetic source of variance and/or a degree of insensitivity of our mapping analysis. Nonetheless, the hypothermia and LORR QTLs may be amenable to refinement (e.g., with introgressed-congenic strategies) and experimental validation (e.g., via building convergent evidence across studies), although this will be complicated by our finding that these phenotypes also showed main effects (hypothermia) or interactions (LORR) with sex.

Our analysis revealed a number of other QTLs that overlap with those previously linked to EtOH-related phenotypes in various mouse populations. For example, a query of the MGD revealed that the hypothermia QTL we found on Chr 7 overlaps *Ethm3* (Crawshaw et al. 2001) and our Chr 2 locus for LORR overlaps with *Alcrsp2* (Erwin et al. 1997). In addition, the Chr 16 hypothermia QTL we found overlaps with that previously linked to similar phenotypes in other mouse populations. This QTL is in the same region as a LORR QTL (Browman and Crabbe 2000) and EtOH drinking phenotype QTL (Gehle and Erwin 1998) previously found in B6 × DBA/2J recombinant inbreds (BXD RI). Interestingly, this same locus has been recently linked to a measure of EtOH AFT in long-sleep/short-sleep mice (Bennett et al. 2007) and EtOH drinking in a B6 × C3H/HeJ F2 population (Drews et al. 2010).

Of the other QTLs we found, loci for BEC at LORR recovery on Chrs 9 and 11 overlap with a number of preference-related loci (Bachmanov et al. 2002; Bice et al. 2006; Erwin et al. 1997; Malmanger et al. 2006; Melo et al. 1996; Phillips et al. 1994; Tarantino et al. 1998), and the Chr 9 locus also overlaps with loci for acute alcohol locomotor activation (Erwin et al. 1997; Malmanger et al. 2006) and conditioned taste aversion (Risinger et al. 1998) and our locus for ataxia. The other locus we found for ataxia on Chr 8 does not overlap any previously discovered alcohol-related loci, but interestingly, it does overlap *Cbm2*, a QTL for cerebellum weight (Airey et al. 2001). Likewise, our QTLs for LORR overlap with alcohol-drinking loci on Chrs 2, 4, 8, and 12 (Bachmanov et al. 2002; Belknap et al. 1997; Bice et al. 2006; Fernandez et al. 1999; Gill and Boyle 2005; Melo et al. 1996; Phillips et al. 1994; Tarantino et al. 1998). Also of particular note is the large number of traits that we found to map to Chr 2, given previous reports that a locus in this region has been linked to various EtOH-related traits in various mouse lines (Crabbe et al. 1994; Gill and Boyle 2005; Malmanger et al. 2006). Candidate gene studies implicate *Stxbp1* as a candidate for consumption-related traits at this locus (Fehr et al. 2005). More broadly, the finding that our LORR QTL overlapped with regions consistently linked to EtOH drinking suggests a common genetic influence on these behaviors. This provides important, albeit indirect, evidence supporting the hypothesis that variation in sensitivity to high-dose (aversive) EtOH intoxication is a factor driving the propensity to drink and, by extension, relative risk for alcohol abuse (Krystal et al. 2003).

The convergent loci across studies could facilitate the reduction of positional candidates using a multiple-cross mapping strategy or other comparison of strain haplotypes, or through the integration of other functional studies. For this reason, we have deposited all QTL positional candidates into the GeneWeaver database (Baker et al. 2011). Using this system, we have identified priority candidates for the Chr 3 EtOH-induced hypothermia locus that may influence multiple EtOH-related responses. The most highly connected candidate is *Hs2st1*, a heparin sulfate sulfotransferase. A search of the Allen Brain Atlas reveals that this gene is highly expressed in the hippocampus. A GeneNetwork query reveals that it is coexpressed with alcohol preference in BXD RI strains. Another compelling candidate is *Prkacb*, interesting because of the already known role of the protein kinase A pathway in both LORR and hypothermia (Yang et al. 2003).

There are several instances where our data do not correspond to prior studies. For example, with the exception of QTLs on Chr 6 previously found for ethanol consumption in a B6.BALB/cJ-introgressed line (Vadasz et al. 2007) and for ethanol-induced locomotor activity (Downing et al. 2003), the LORR QTLs we report are largely distinct from those reported for LORR duration in BXD RI (Browman and Crabbe 2000), LXS (Haughey et al. 2005), and long-sleep/short-sleep mice (Bennett et al. 2002, 2008) populations, EtOH drinking in BXDs (Phillips et al. 1998), as well as EtOH drinking (Belknap et al. 1997; McClearn et al. 1997) and EtOH-induced locomotor stimulation in a B6 × D2 intercross (Hitzemann et al. 1998).

QTL mapping studies rarely have sufficient power to reveal all causative loci underlying complex phenotypes, and in our study we also failed to reproduce the QTL on a Chr 1 “hotspot” previously linked to multiple EtOH phenotypes in other mouse crosses (Ehlers et al. 2010; Mozhui et al. 2008). These situations could be the result of differences in segregating alleles in each of these populations and heterozygosity in our B6 × S1 F2 population, methodological differences in the measurement of EtOH-related phenotypes between studies, or simply genuine false negatives. Although we performed genome-wide searches, it is computationally prohibitive to search the entire multiple-locus model space. Future studies using alternative statistical QTL models may have better fidelity. It will also be important to take some of our provisional findings further by testing for convergent evidence from other crosses.

A consistent finding in the QTL–EtOH literature that was also a major pattern in our data is the largely nonoverlapping QTLs across phenotypes (e.g., see Browman and Crabbe 2000; Drews et al. 2010; Gehle and Erwin 1998; Phillips et al. 1998). This was echoed by our phenotypic correlational analysis, which found few significant correlations between phenotypes. One exception was a significant negative correlation between ataxia and LORR, indicating that high sensitivity to EtOH’s ataxic effects predicted high sensitivity to LORR. Longer LORR time was also associated with lower BECs on awakening. This is a general relationship and is not unexpected if LORR duration is a function of EtOH clearance as opposed to being modulated independently of LORR time by, for example, pharmacokinetic factors. Nonetheless, both measures can provide useful measures of EtOH sensitivity and are best considered together. The more general conclusion from these correlational analyses across the various end-point measures we made is that the pattern of largely noncorrelations is consistent with largely independent genetic influences.

In conclusion, the current study found a number of genomic locations associated with three different behavioral measures of EtOH intoxication. The most compelling QTLs were identified for hypothermia and LORR, with provisional QTLs found for ataxia. The hypothermia and LORR QTLs were found at separate genomic regions, suggesting predominantly distinct genetic contributions to these measures of intoxication. Current data provide a basis for further studies, which by utilizing sequence data, gene expression repositories, QTL archives, and integrative functional genomic tools could identify specific polymorphisms within these QTLs. In the longer term, uncovering the candidate genes associated with variation in these phenotypes in this mouse population could provide novel insight into genetic factors that might also underlie sensitivity to the negative feelings of EtOH intoxication in humans.

## Electronic supplementary material

Below is the link to the electronic supplementary material.

Supplementary material 1 (DOC 79 kb)

Supplementary Fig. 1 Average rotarod training latency. **A** S1 and F2 had a lower latency to fall than B6. **B** Frequency histogram illustrates normal distribution (dashed line is normal distribution) of training index scores in the F2 population. **C** A scan for single-locus main effects found several suggestive and significant loci (black) and a scan for sex-interacting loci found a locus on Chr 5. An additional locus on distal 7 may also interact with sex. **D** Effect plots for each of the main effects and a sex-interacting locus; *P < 0.05. Data are mean ± SEM (EPS 1228 kb)

Supplementary Fig. 2 Rotarod training index. **A** S1 had a higher training index (increase in latency to fall from training trial 1 to trial 10) than F2 or F1. **B** Frequency histogram illustrates normal distribution (dashed line is normal distribution) of training index scores in the F2 population. **C** A scan for single-locus main effects found a suggestive locus on Chr 11. **D** Effect plot for the peak locus on Chr 11; *p < 0.05. Data are mean ± SEM (EPS 1111 kb)
