# Supplementary material for: Quantitative trait loci for sensitivity to ethanol intoxication in a C57BL/6J × 129S1/SvImJ inbred mouse cross
Source: Mamm Genome. 2012 Feb 28;23(5):305–21. doi: 10.1007/s00335-012-9394-2 (PMC3357470; doi:10.1007/s00335-012-9394-2)
Supplement: Supplementary file 1 — Supplementary material 1 (DOC 79 kb) [file 335_2012_9394_MOESM1_ESM.doc]

| Supplemental Table 1: Intersection of QTLs with previously reported loci for alcohol related phenotypes | | | | |
| --- | --- | --- | --- | --- |
| Each 1.5 LOD confidence interval was queried for all QTLs in the Mouse Genome Database. All alcohol related measures are reported below. Two QTLs replicate loci previously reported for similar traits and are **bolded.** For another two of the loci no alcohol related QTLs were found. Related neurobiological measures for these traits are shown in red. Additional loci not present in the database may also overlap. | | | | |
|  |  |  |  |  |
| Chr | Measure | QTL | Trait | Reference |
| 3 | alcohol induced hypothermia | *Alcp3* | alcohol preference locus 3, male specific | J:72842, Belknap JK; Atkins AL, The replicability of QTLs for murine alcohol preference drinking behavior across eight independent studies., Mamm Genome 2001 Dec;12(12):893-9 |
|  |  | *Ap6q* | alcohol preference 6 QTL | J:49707 Tarantino LM et al., "Confirmation of quantitative trait loci for alcohol preference in mice." Alcohol Clin Exp Res 1998 Aug;22(5):1099-105 |
|  |  | *Letohc1* | low ethanol consumption 1 | J:40854 Belknap JK et al., "Short-term selective breeding as a tool for QTL mapping: ethanol preference drinking in mice." Behav Genet 1997 Jan;27(1):55-66 |
|  |  | *Lore10* | loss of righting induced by ethanol 10 | J:114558 Bennett B et al., "Confirmation and fine mapping of ethanol sensitivity quantitative trait loci, and candidate gene testing in the LXS recombinant inbred mice." J Pharmacol Exp Ther 2006 Oct;319(1):299-307 |
| 7 | alcohol induced hypothermia | *Alcp12* | alcohol preference locus 12, male specific | J:52467 Gill K et al., "Alcohol preference in AXB/BXA recombinant inbred mice: gender differences and gender-specific quantitative trait loci." Mamm Genome 1998 Dec;9(12):929-35 |
|  |  | *Alcp14* | alcohol preference locus 14, female specific | J:52467 Gill K et al., "Alcohol preference in AXB/BXA recombinant inbred mice: gender differences and gender-specific quantitative trait loci." Mamm Genome 1998 Dec;9(12):929-35 |
|  |  | *Ap7q* | alcohol preference 7 QTL | J:78215 Bachmanov AA et al., "Voluntary ethanol consumption by mice: genome-wide analysis of quantitative trait loci and their interactions in a C57BL/6ByJ x 129P3/J F2 intercross." Genome Res 2002 Aug;12(8):1257-68 |
|  |  | ***Ethm3*** | **ethanol induced thermoregulation 3** | J:73579 Crawshaw LI et al., "Influence of ethanol on thermoregulation: mapping quantitative trait loci." Physiol Genomics 2001 Dec 21;7(2):159-69 |
| 16 | alcohol induced hypothermia | *Afteq2* | acute functional tolerance to ethanol QTL 2 | J:122921 Bennett B et al., "Quantitative trait locus mapping for acute functional tolerance to ethanol in the L x S recombinant inbred panel." Alcohol Clin Exp Res 2007 Feb;31(2):200-8 |
| 2 | ethanol induced LORR | *Actre2* | activity response to ethanol 2 | J:71081 Demarest K et al., "Further characterization and high-resolution mapping of quantitative trait loci for ethanol-induced locomotor activity." Behav Genet 2001 Jan;31(1):79-91 |
|  |  | *Actre3* | activity response to ethanol 3 | J:71081 Demarest K et al., "Further characterization and high-resolution mapping of quantitative trait loci for ethanol-induced locomotor activity." Behav Genet 2001 Jan;31(1):79-91 |
|  |  | *Actre4* | activity response to ethanol 4 | J:71081 Demarest K et al., "Further characterization and high-resolution mapping of quantitative trait loci for ethanol-induced locomotor activity." Behav Genet 2001 Jan;31(1):79-91 |
|  |  | *Alcp1* | alcohol preference locus 1, male specific | J:33277 Melo JA et al., "Identification of sex-specific quantitative trait loci controlling alcohol preference in C57BL/ 6 mice." Nat Genet 1996 Jun;13(2):147-53 |
|  |  | ***Alcrsp2*** | **alcohol response 2** | J:39049 Erwin VG et al., "Common quantitative trait loci for alcohol-related behaviors and central nervous system neurotensin measures: hypnotic and hypothermic effects." J Pharmacol Exp Ther 1997 Feb;280(2):911-8 |
|  |  | *Alcw4* | alcohol withdrawal 4 | J:40120 Crabbe JC et al., "Use of recombinant inbred strains for studying genetic determinants of responses to alcohol." Alcohol Alcohol Suppl 1994;2():67-71 |
|  |  | *Alpq2* | alcohol preference QTL 2 | J:110926 Bice PJ et al., "Identification of QTLs influencing alcohol preference in the High Alcohol Preferring (HAP) and Low Alcohol Preferring (LAP) mouse lines." Behav Genet 2006 Mar;36(2):248-60 |
|  |  | *Ap2q* | alcohol preference 2 QTL | J:49707, Tarantino LM; McClearn GE; Rodriguez LA; Plomin R, Confirmation of quantitative trait loci for alcohol preference in mice., Alcohol Clin Exp Res 1998 Aug;22(5):1099-105 |
|  |  | *Elda* | ethanol induced low dose activation 3 | J:114560 Downing C et al., "QTL mapping for low-dose ethanol activation in the LXS recombinant inbred strains." Alcohol Clin Exp Res 2006 Jul;30(7):1111-20 |
|  |  | *etax3* | ethanol induced ataxia 3 | J:78665 Kirstein SL et al., "Quantitative trait loci affecting initial sensitivity and acute functional tolerance to ethanol-induced ataxia and brain cAMP signaling in BXD recombinant inbred mice." J Pharmacol Exp Ther 2002 Sep;302(3):1238-45 |
|  |  | *Etohc1* | ethanol consumption 1 | J:40854 Belknap JK et al., "Short-term selective breeding as a tool for QTL mapping: ethanol preference drinking in mice." Behav Genet 1997 Jan;27(1):55-66 |
|  |  | *Etohc2* | ethanol consumption 2 | J:20482 Phillips TJ et al., "Localization of genes affecting alcohol drinking in mice." Alcohol Clin Exp Res 1994 Aug;18(4):931-41 |
|  |  | *Etohila* | ethanol induced locomotor activity | J:52087 Hitzemann R et al., "Genetics of ethanol-induced locomotor activation: detection of QTLs in a C57BL/6J x DBA/2J F2 intercross." Mamm Genome 1998 Dec;9(12):956-62 |
|  |  | *Etohr* | ethanol response acute | J:52042 Demarest K et al., "Identification of an acute ethanol response quantitative trait locus on mouse chromosome 2." J Neurosci 1999 Jan 15;19(2):549-61 |
|  |  | *Vacq3* | voluntary alcohol consumption QTL 3 | J:100637 Gill K et al., "Genetic analysis of alcohol intake in recombinant inbred and congenic strains derived from A/J and C57BL/6J progenitors." Mamm Genome 2005 May;16(5):319-31 |
|  |  | *Vacq4* | voluntary alcohol consumption QTL 4 | J:100637 Gill K et al., "Genetic analysis of alcohol intake in recombinant inbred and congenic strains derived from A/J and C57BL/6J progenitors." Mamm Genome 2005 May;16(5):319-31 |
| 4 | ethanol induced LORR | *Ap3q* | alcohol preference 3 QTL | J:49707 Tarantino LM et al., "Confirmation of quantitative trait loci for alcohol preference in mice." Alcohol Clin Exp Res 1998 Aug;22(5):1099-105 |
| 8 | ethanol induced LORR | *Alcw6* | alcohol withdrawal 6 | J:84090 Bergeson SE et al., "Chromosomal loci influencing chronic alcohol withdrawal severity." Mamm Genome 2003 Jul;14(7):454-63 |
|  |  | *Apq8* | alcohol preference 8 QTL | J:78215 Bachmanov AA et al., "Voluntary ethanol consumption by mice: genome-wide analysis of quantitative trait loci and their interactions in a C57BL/6ByJ x 129P3/J F2 intercross." Genome Res 2002 Aug;12(8):1257-68 |
| 12 | ethanol induced LORR | *Aaq2* | alcohol acceptance QTL 2, female specific | J:67846 Fernandez JR et al., "Sex-exclusive quantitative trait loci influences in alcohol-related phenotypes." Am J Med Genet 1999 Dec 15;88(6):647-52 |
| 19 | ethanol induced LORR | *N/A* | Nearest hit: *Stnn*, striatal neuron number | J:69683 Rosen GD et al., "Complex trait analysis of the mouse striatum: independent QTLs modulate volume and neuron number." BMC Neurosci 2001;2(1):5 |
| 6 | ethanol induced LORR-2 part | *Eila2* | ethanol induced locomotor activity 2 | J:85747 Downing C et al., "Genetic analysis of the psychomotor stimulant effect of ethanol." Genes Brain Behav 2003 Jun;2(3):140-51 |
| 7 | LORR BEC | *Ap7q* | alcohol preference 7 QTL | J:78215 Bachmanov AA et al., "Voluntary ethanol consumption by mice: genome-wide analysis of quantitative trait loci and their interactions in a C57BL/6ByJ x 129P3/J F2 intercross." Genome Res 2002 Aug;12(8):1257-68 |
| 9 | LORR BEC | *Actre6* | activity response to ethanol 6 | J:116204 Malmanger B et al., "Further studies on using multiple-cross mapping (MCM) to map quantitative trait loci." Mamm Genome 2006 Dec;17(12):1193-204 |
|  |  | *Alcrsp4* | alcohol response 4 | J:39049 Erwin VG et al., "Common quantitative trait loci for alcohol-related behaviors and central nervous system neurotensin measures: hypnotic and hypothermic effects." J Pharmacol Exp Ther 1997 Feb;280(2):911-8 |
|  |  | *Alpq1* | alcohol preference QTL 1 | J:110926 Bice PJ et al., "Identification of QTLs influencing alcohol preference in the High Alcohol Preferring (HAP) and Low Alcohol Preferring (LAP) mouse lines." Behav Genet 2006 Mar;36(2):248-60 |
|  |  | *Alpq3* | alcohol preference QTL 3 | J:110926 Bice PJ et al., "Identification of QTLs influencing alcohol preference in the High Alcohol Preferring (HAP) and Low Alcohol Preferring (LAP) mouse lines." Behav Genet 2006 Mar;36(2):248-60 |
|  |  | *Alpq5* | alcohol preference 5 QTL | J:49707, Tarantino LM; McClearn GE; Rodriguez LA; Plomin R, Confirmation of quantitative trait loci for alcohol preference in mice., Alcohol Clin Exp Res 1998 Aug;22(5):1099-105 |
|  |  | *Etohc3* | ethanol consumption 3 | J:20482, Phillips TJ; Crabbe JC; Metten P; Belknap JK, Localization of genes affecting alcohol drinking in mice., Alcohol Clin Exp Res 1994 Aug;18(4):931-41 |
|  |  | *Etohcta8* | ethanol conditioned taste aversion 8 | J:50271 Risinger FO et al., "Ethanol-induced conditioned taste aversion in BXD recombinant inbred mice." Alcohol Clin Exp Res 1998 Sep;22(6):1234-44 |
| 11 | LORR BEC | *Alcp2* | alcohol preference locus 2, female specific | J:33277 Melo JA et al., "Identification of sex-specific quantitative trait loci controlling alcohol preference in C57BL/ 6 mice." Nat Genet 1996 Jun;13(2):147-53 |
| 8 | ETOH ataxia | *N/A* | Nearest hit: *Cbm2*, cerebellum weight | J:70179 Airey DC et al., "Genetic control of the mouse cerebellum: identification of quantitative trait loci modulating size and architecture." J Neurosci 2001 Jul 15;21(14):5099-109 |
| 9 | ETOH ataxia | *Actre6* | activity response to ethanol 6 | J:116204 Malmanger B et al., "Further studies on using multiple-cross mapping (MCM) to map quantitative trait loci." Mamm Genome 2006 Dec;17(12):1193-204 |
|  |  | *Alcrsp4* | alcohol response 4 | J:39049 Erwin VG et al., "Common quantitative trait loci for alcohol-related behaviors and central nervous system neurotensin measures: hypnotic and hypothermic effects." J Pharmacol Exp Ther 1997 Feb;280(2):911-8 |
|  |  | *Alpq1* | alcohol preference QTL 1 | J:110926 Bice PJ et al., "Identification of QTLs influencing alcohol preference in the High Alcohol Preferring (HAP) and Low Alcohol Preferring (LAP) mouse lines." Behav Genet 2006 Mar;36(2):248-60 |
|  |  | *Alpq3* | alcohol preference QTL 3 | J:110926 Bice PJ et al., "Identification of QTLs influencing alcohol preference in the High Alcohol Preferring (HAP) and Low Alcohol Preferring (LAP) mouse lines." Behav Genet 2006 Mar;36(2):248-60 |
|  |  | *Alpq5* | alcohol preference 5 QTL | J:49707, Tarantino LM; McClearn GE; Rodriguez LA; Plomin R, Confirmation of quantitative trait loci for alcohol preference in mice., Alcohol Clin Exp Res 1998 Aug;22(5):1099-105 |
|  |  | *Etohc3* | ethanol consumption 3 | J:20482, Phillips TJ; Crabbe JC; Metten P; Belknap JK, Localization of genes affecting alcohol drinking in mice., Alcohol Clin Exp Res 1994 Aug;18(4):931-41 |
|  |  | *Etohcta8* | ethanol conditioned taste aversion 8 | J:50271 Risinger FO et al., "Ethanol-induced conditioned taste aversion in BXD recombinant inbred mice." Alcohol Clin Exp Res 1998 Sep;22(6):1234-44 |
